# Balancing autonomy and beneficence at the time of psychiatric discharge

**DOI:** 10.1186/s13584-017-0201-0

**Published:** 2018-01-02

**Authors:** Abhishek Jain, Paul S. Appelbaum

**Affiliations:** Department of Psychiatry, Columbia University, and New York State Psychiatric Institute, 1051 Riverside Dr., New York, NY 10032 USA

**Keywords:** Autonomy, Beneficence, Civil commitment, Discharge, Ethics, Involuntary hospitalization, Judicial review, Law, Non-medical decision-making, Psychiatry

## Abstract

As in much of the world, mental health law in Israel has evolved over the past half-century toward greater protection of patients’ liberty and an increased emphasis on due process. Part of that process in Israel involved taking decisions about prolonged involuntary hospitalization out of the hands of treating psychiatrists and turning them over to independent review panels. Argo and colleagues examined outcomes of discharge decisions made by these panels compared with treating psychiatrists. In this brief commentary, we describe related trends in mental health law in other countries, especially the U.S., consider countervailing perspectives on the role of review panels, and suggest how the Argo et al. study might be followed up with additional research.

## Background

Argo and colleagues retrospectively examined outcomes of psychiatric hospital discharge decisions made by district psychiatric committees (DPCs) compared to treating psychiatrists (TPs), based on data from the Ministry of Health’s National Psychiatric Hospitalization Registry. [1] They concluded that patients discharged by DPCs (which have a magistrate-level attorney as chair and two psychiatrists as members) had a higher probability of readmission than patients discharged by TPs. Based on their data, they suggest that judicial decisions are driven by faulty assumptions that patients will fare better in the community than in the hospital.

A number of authors have described the evolution of Israel’s mental health law and its shift over the past half-century towards greater emphasis on patient liberty and autonomy, and on community treatment [2–4]. In this brief commentary, we describe similar trends in other countries, especially the U.S., consider countervailing perspectives, and suggest how Argo and colleagues’ study might lead to additional research.

### Evolution of civil commitment law

The development of Israel’s civil commitment law mirrors trends seen elsewhere, including in the U.S. From the earliest days of psychiatric hospitalization in the U.S., admission and discharge decisions were in the hands of physicians, who determined whether patients needed hospital treatment. It was, in essence, a beneficence-based approach. The introduction of involuntary commitment laws in the early nineteenth-century formalized that process, but it was not until later in the century that most commitment decisions received routine judicial review. In the mid-twentieth century, however, questions about the benefits of hospitalization, arguments for community-based treatment (facilitated by the emergence of novel psychopharmacologic treatments), greater respect for the civil liberties of people with mental illness, and a desire for more cost-effective care precipitated changes in the process [5].

By the end of the 1970s, involuntary commitment laws in every state in the U.S. were revised. Commitment was limited to persons who were likely to be a danger to themselves or others, or unable to care for themselves. Patients seeking to challenge their involuntary hospitalization were afforded greater protections, often drawn from procedures for criminal defendants, including rights to notice of the hearing, to testify and call witnesses, and to appeal adverse findings. Other countries - including countries as diverse as Canada, Germany, Taiwan, and of course Israel - followed suit [6]. The reforms of the 1970s remain largely in place around the world today.

However, there are often substantial variations across jurisdictions, and Israeli law differs from statutes in the U.S. in a number of ways. In Israel, involuntary hospitalization is predicated on the patient being psychotic [7], which is not a requirement in the U.S. Whereas the majority of members of Israel’s District Psychiatric Committees, which review requests for extended commitment, are psychiatrists, in the U.S. similar requests are reviewed by judges or hearing officers, typically with no clinical training. In addition, mean length of psychiatric hospitalization generally has been longer in Israel (36 days [8] vs. 7 to 13 days in the U.S. [9, 10]), where facilities have not been subject to the same degree of vigorous utilization management as U.S. hospitals.

Notwithstanding the differences, concerns similar to the ones expressed by Argo et al. about the consequences of someone other than the treating psychiatrist making discharge decisions have been raised by psychiatrists in the U.S. and other countries. Opponents of U.S. civil commitment reforms in the last century argued that reducing the reliance of judges on the opinions of treating psychiatrists—a predicted consequence of the embrace of dangerousness standards for involuntary commitment—would deprive patients of needed treatment. However, taken in the aggregate, studies have failed to demonstrate consistent changes in the rates of commitment or the characteristics of committed patients, or evidence that patients in need of immediate hospitalization were being turned away, as direct consequences of changes in the laws [5].

We think that it is fair to say that most psychiatrists in the U.S., Canada, Australia, and Western Europe, where decisions about extending involuntary hospitalization are in judicial or quasi-judicial hands, have reconciled themselves to the process. Indeed, many clinicians may have come to recognize advantages to a court-driven process, such as maintaining an alliance with the patient when someone else makes the discharge decision [11]. Perhaps surprisingly, studies have suggested that rather than strictly applying dangerousness standards to their release decisions, judges may actually follow more of a *parens patriae* approach, taking into account patients’ treatment needs, the availability of community-based services [12], and the impact on families of premature discharges [13]. Moreover, even in dangerousness-based determinations, judges frequently defer to medical recommendations [12, 14].

One other trend worth noting is the development of outpatient civil commitment, sometimes referred to as Assisted Outpatient Treatment (AOT) or, in the British Commonwealth, Community Treatment Orders (CTO). New York’s statute, passed in 1999, is typical of the newer generation of outpatient commitment laws in American states. Usually applied to patients at the time of hospital discharge, it focuses on patients who have repeatedly failed to adhere to treatment, resulting in hospitalization or arrest. After a court hearing, patients may be required to receive regular psychiatric care, take medications, participate in rehabilitation programs, and avoid substance use [15]. Outpatient commitment laws allow patients greater liberty than inpatient commitment, while still requiring compliance with treatment, balancing liberty and beneficence. Currently, 45 states and the District of Columbia have such statutes, which studies suggest are associated with increased access to care, improved adherence to treatment, reduced costs, fewer hospitalizations, and decreased risk of arrest [16–18].

### Autonomy, beneficence and involuntary commitment

Changes to commitment laws around the world have been driven by desires to restrict the grounds for deprivation of liberty and guarantee independent review of such deprivations when they occur. In the U.S., these reforms were based on new understandings of constitutional limitations on state power and requirements for due process [5]; elsewhere, they often have been based on notions of intrinsic human rights to liberty and autonomy [19]. The current generation of involuntary commitment laws typically requires periodic reviews of the status of committed patients, which focus on whether commitment criteria continue to be met, and places the decision in the hands of judges or panels exercising quasi-judicial functions. It seems evident that contemporary civil commitment processes are designed to protect fundamental rights to liberty and autonomy, even at the risk of less effective treatment, more rapid decompensation, and higher rates of re-hospitalization. In these processes, rights are given precedence over treatment considerations: beneficence is subordinated to liberty.

How great are the costs of taking these discharge decisions out of the hands of treating psychiatrists, a nearly universal characteristic of modern laws? Civil liberties advocates might note that Argo et al.’s data suggest the cost is not particularly great. Patients discharged by DPCs remained in the community for a median of 175 days compared to 211 days for the TP group, a difference of 36 days. But patients discharged by TPs were kept more than twice as long in the hospital (76 days vs. 30 days), a difference of 46 days, not only wiping out the advantage of time in the community for TP decisions, but apparently reversing it. Moreover, the differences in “failure” rate between the groups, as defined by the researchers (see below), were decidedly modest. From this perspective, DPCs not only offer a check on the unbridled discretion of treating psychiatrists that protects patients’ rights to liberty, they do so at no cost (and perhaps some benefit) to length of time in the community.

### Moving forward with a research agenda

Argo and colleagues found statistically significant differences in rates of readmission between DPC and TP discharged patients, including an adjusted 30% lower probability of “failure” (defined as readmission in less than 30 days, involuntary civil readmission in less than 180 days, and involuntary readmission under court order in less than one year) in the TP group. However, the absolute difference in readmission rates, though statistically significant, was somewhat modest (4.6%, 7%, and 6.4% within 30, 180 and 365 days, respectively) and varied markedly among groups by age, sex, diagnosis, and duration of index hospitalization. Nor do these data address the possibility that discharged patients do better in other ways (e.g., maintaining housing or employment). Thus, future comparisons of discharge decisions by treaters and review panels or judges may benefit from additional outcome measures, such as measures of symptoms, rates of adherence to aftercare and medications, and social outcomes, and from the use of validated rating scales. Although readmission rates may be a valid quality measure of psychiatric hospitalization, other methods may provide a finer-grained basis for comparison [20].

Further elucidating the differences between DPC and TP decision processes (and their equivalents in other countries) would also be informative. What additional factors associated with good outcomes in the community (e.g., availability of community services, previous engagement in outpatient treatment, presence of supportive family members and friends) may help to explain disagreement between DPCs and TPs? Future investigators might also explore the extent to which TPs seek renewed involuntary commitment because of liability concerns, not a belief that patients continue to meet commitment criteria or would benefit from prolonged hospital stays. That is, since DPCs are immunized from liability whereas TPs are not [21], are some cases taken to the committee knowing that patients will be released, because the treating psychiatrist is reluctant to assume the risk of authorizing discharge? Pertinent to the Israeli context, in a recent survey of 213 Israeli psychiatry residents and psychiatrists, 62% reported practicing defensive psychiatry, and 54% specifically acknowledged advising unwarranted hospitalization of suicidal patients [22]. To the extent that treating psychiatrists themselves would discharge patients in the absence of a DPC mechanism, inclusion of such cases confounds examination of instances in which their decisions would differ.

## Conclusions

Argo and colleagues provide a useful starting point for examining the effects of policies relegating decisions about prolonged involuntary hospitalization to quasi-judicial panels like the DPCs. Although the mandate of such review bodies is not to maximize patients’ clinical benefit—liberty trumps beneficence under most contemporary statutes—exploration in other jurisdictions of the consequences of their decisions is warranted. Perhaps, as here, the data that result will be unexpectedly reassuring.
